# Time Dependent Dielectric Breakdown in Copper Low-k Interconnects: Mechanisms and Reliability Models

**DOI:** 10.3390/ma5091602

**Published:** 2012-09-12

**Authors:** Terence K.S. Wong

**Affiliations:** Division of Microelectronics, School of Electrical and Electronic Engineering, Nanyang Technological University, Block S2, Nanyang Avenue, Singapore 639798, Singapore; E-Mail: ekswong@ntu.edu.sg; Tel.: +65-67906401; Fax: +65-67933318.

**Keywords:** low-k dielectric, interconnect dielectric breakdown, soft breakdown, reliability

## Abstract

The time dependent dielectric breakdown phenomenon in copper low-k damascene interconnects for ultra large-scale integration is reviewed. The loss of insulation between neighboring interconnects represents an emerging back end-of-the-line reliability issue that is not fully understood. After describing the main dielectric leakage mechanisms in low-k materials (Poole-Frenkel and Schottky emission), the major dielectric reliability models that had appeared in the literature are discussed, namely: the Lloyd model, *1/E* model, thermochemical *E* model, *E^1/2^* models, *E^2^* model and the Haase model. These models can be broadly categorized into those that consider only intrinsic breakdown (Lloyd, *1/E*, *E* and Haase) and those that take into account copper migration in low-k materials (*E^1/2^*, *E^2^*). For each model, the physical assumptions and the proposed breakdown mechanism will be discussed, together with the quantitative relationship predicting the time to breakdown and supporting experimental data. Experimental attempts on validation of dielectric reliability models using data obtained from low field stressing are briefly discussed. The phenomenon of soft breakdown, which often precedes hard breakdown in porous ultra low-k materials, is highlighted for future research.

## 1. Introduction

Since the beginning of the 21st century, the semiconductor industry has adopted low dielectric constant (low-k) dielectrics as the insulating material in multi-level copper damascene interconnects for ultra large-scale integration (ULSI) integrated circuits (IC) [1]. Low-k dielectrics with dielectric constants lower than that of conventional silicon oxide (k = 4.0) are needed in order to reduce the resistance-capacitance delay (or latency), crosstalk and dynamic power dissipation in the interconnect stack. These interconnect-related parasitic phenomena had become the main performance limiters in ULSI ICs. More recently, ultra low-k dielectrics with a porous microstructure had been integrated with copper metallization. Examples of low-k dielectrics include: fluorinated silicon oxide (SiOF) [2], carbon doped silicon oxide (SiOCH) [3,4], spin-on methyl-silisesquioxanes (MSQ) [5] and organic polymers [6]. The dielectric constants of these materials are reduced, relative to silicon oxide by incorporation of smaller and more electronegative atoms or space-filling functional groups (e.g., -CH_3_). Starting with a low-k dielectric matrix, an ultra low-k version of the same material can be obtained by using a sacrificial porogen which is co-deposited with the matrix material and then subsequently pyrolyzed *in-situ* [7,8]. The porogen acts as a template for the nanoscale pores that are formed after pyrolysis. The voids within an ultra low-k dielectric reduce the mass density of the material and the dielectric constant. The deposition methods, synthesis techniques, material properties and characterization of low-k and ultra low-k dielectrics had been the subject of numerous reviews in the past decade and will not be repeated here [9,10,11,12,13,14]. However, it is pertinent to point out an early key observation that with the reduction of the dielectric constant k, there is a tendency for the mechanical, thermal, electrical and adhesion properties of the low-k dielectrics to become inferior with respect to silicon oxide [10]. This is significant because it implies that an interconnect structure with integrated low-k and ultra low-k dielectrics is potentially vulnerable to reliability issues and new failure mechanisms may be observed.

In this article, we provide an up-to-date review on the time dependent dielectric breakdown (TDDB) phenomenon in low-k and ultra low-k dielectrics in damascene copper interconnects. Unlike previous reviews on low-k materials, a review on low-k TDDB reliability is currently lacking in the literature. This topic is also not mentioned in recent reference books on semiconductor device failure mechanisms [15]. However, as highlighted in the 2009 International Roadmap for Semiconductors (ITRS) [16], low-k TDDB is an integrated circuit reliability phenomenon that is of growing significance. Despite much research, there is insufficient basic understanding, and this poses a future technical challenge. It is therefore useful to survey the present state of knowledge of this problem. In general, TDDB refers to the catastrophic loss of the insulating properties of a dielectric when it is subjected to voltage/current bias and temperature stress. TDDB is most often (but not always) manifested as an abrupt and irreversible increase in the leakage current when the sample is under constant bias stress at elevated temperature. It is also referred to as hard breakdown (HBD). In the past, TDDB was primarily a front end-of-the-line (FEOL) issue because of the high fields in the thin gate oxide of metal oxide semiconductor field effect transistors (MOSFETs). For interconnects, TDDB had not been a concern until recent years, because the silicon oxide used for inter- and intra-level dielectric is dense and has excellent insulating properties. In addition, the applied electric fields were much lower than those in the thin gate oxide. With the advent of low-k and ultra low-k dielectrics and aggressive scaling, however, the situation has changed completely. Due to the inferior dielectric breakdown strength and the nanoscale half pitch spacing in present ULSI local interconnects, the electric fields within the low-k materials can approach 1MV/cm. This increases the likelihood of low-k TDDB and makes it an important reliability problem for nanoscale ICs.

Since 2003, many dielectric reliability models have been proposed to explain and predict the TDDB phenomenon in low-k dielectrics under electrical bias stress. This is an indication that the state of understanding of this field is far more limited than that for thin gate oxide (SiO_2_) TDDB in the 1990s. (Thin gate oxide refers to thermal SiO_2_ with a thickness generally between 4 nm and 10 nm.) For the gate oxide integrity problem, there were three physical models, namely the thermochemical (or *E*) model [17,18] and the *1/E* model [17] for thin oxides and the power law model (*1/V^n^*) for ultrathin (sub-4 nm) oxides where *V* is the voltage across the gate oxide and *n* is an exponent [19]. On the other hand, for the low-k TDDB phenomenon, eight dielectric reliability models had been proposed by various research groups [20,21,22,23,24,25,26,27]. Although experimental data was presented for each model that showed support for the proposed TDDB mechanism, no low-k TDDB model so far has been able to predict low field time to failure (TTF) for a wide range of low-k dielectrics. It is important to have a correct understanding of the failure mechanism because each model predicts a different relationship between the TTF and the electric field and temperature. In order to evaluate a new low-k dielectric with lower dielectric constant, the performance at operational electric fields has to be extrapolated from the TTF measured during accelerated testing where higher fields are applied. This extrapolation is based on the important assumption that the mechanism of failure at high and low fields is identical. If the TTF *versus* electric field relationship is inaccurate, the predicted TTF at operational conditions could be too conservative, or worse still, too optimistic.

This review paper is structured as follows. First, the main leakage current mechanisms in low-k materials are discussed. This will be followed by the Lloyd model [20], *1/E* model [21], the thermochemical *E* model [22], Haase model [23], Wu model [24], the *E^1/2^* models [25,26] and the *E^2^* model [27]. For clarity, these models are organized into intrinsic models (Section 3) and extrinsic models (Section 4). The *1/E* and the thermochemical models are the same as those used for describing TDDB in thin gate oxides. The power law model which had not been applied to low-k TDDB will not be discussed. For each dielectric reliability model, the basic assumptions and the proposed underlying physical mechanism will be explained. The derivation of the TTF *versus* field relationship from the physical mechanism will then be summarized and experimental data in support of the model will be discussed. The review will be followed by a section on recent experiments comparing the various models when they are applied to TDDB data from the same set of Cu low-k test structures. A brief discussion of soft breakdown in ultra low-k dielectrics is given before the conclusion and outlook.

## 2. Low-k Dielectric Leakage Mechanisms

Before discussing the various low-k TDDB models, it is useful to highlight the main dielectric leakage mechanisms in these materials. This is because in several of these models, current leakage is an integral part of the model. As discussed in [28,29], there are seven known conduction mechanisms in insulating materials. These include: Fowler-Nordheim tunneling, direct (Giaever) tunneling, Schottky emission, Poole-Frenkel emission, ohmic conduction, space-charge-limited conduction and ionic conduction. For low-k dielectrics, the conduction mechanisms that had been experimentally observed are ohmic conduction, Schottky emission and Poole-Frenkel emission. In the following, only the latter two main mechanisms will be discussed.

Since dielectric materials have wide band gaps, their intrinsic carrier concentration is extremely low. For conduction, carriers have to be introduced from external metallic electrodes via a contact. In the Schottky mechanism, electrons are injected from a rectifying contact [28]. The work function of the metal should be greater than the electron affinity of the dielectric and the difference between the two is the barrier height *φ_B_* of the Schottky contact for electrons as seen from the metal. Those electrons in the metal with sufficient energy can surmount the energy barrier and enter the dielectric. The expression for the current density *J_SE_* as a function of the electric field *E* in the dielectric and the absolute temperature *T* can be written as [28]:

(1) $$J_{SE}=A*T^{2}\text{exp}\left[\frac{-q\left(\phi_{B}-\sqrt{qE/4\pi \varepsilon_{0}k}\right)}{k_{B}T}\right]$$

in this equation, A^*^ is the effective Richardson constant; *q* is the electron charge; ε*_0_* is the permittivity of free space; *k* is the dielectric constant and *k_B_* is the Boltzmann constant. The second term inside the exponential function accounts for the image force lowering effect of the emitted electron on the barrier height [28]. The effective barrier height is therefore lower than *φ_B_* and depends on the square root of the ratio of the dielectric field and dielectric constant. Due to the thermal excitation mechanism, the Schottky emission current density is strongly dependent on the absolute temperature. The Schottky barrier height *φ_B_* can be found from a semi-logarithmic plot of ln(*J_SE_/T^2^)* *versus* *E^1/2^*.

The Poole-Frenkel conduction mechanism occurs in dielectrics with intrinsic defects (traps), such as silicon nitride (SiN) and silicon oxynitride (SiON) [30]. These traps are formed during the deposition process and their Coulombic potential can trap electrons. Conduction occurs by field-assisted thermal excitation of electrons from trap to trap [30]. For Poole-Frenkel emission, the barrier height ϕ*_B_* for trapped electrons is given by the energy of the trap relative to the conduction band edge. As with Schottky emission, the barrier height can be lowered by an applied field. The current density in the Poole-Frenkel mechanism is given by [28]:

(2) $$J_{PF}\propto E\text{exp}\left[\frac{-q\left(\phi_{B}-\sqrt{qE/\pi \varepsilon_{0}k}\right)}{k_{B}T}\right]$$

It is pointed out that for Poole-Frenkel emission, the image force lowering effect is twice as strong in comparison with the Schottky mechanism. The barrier height can be found from a semi-logarithmic plot of ln(*J_PF_*/*E*) *versus* *E^1/2^*.

**Figure 1:**
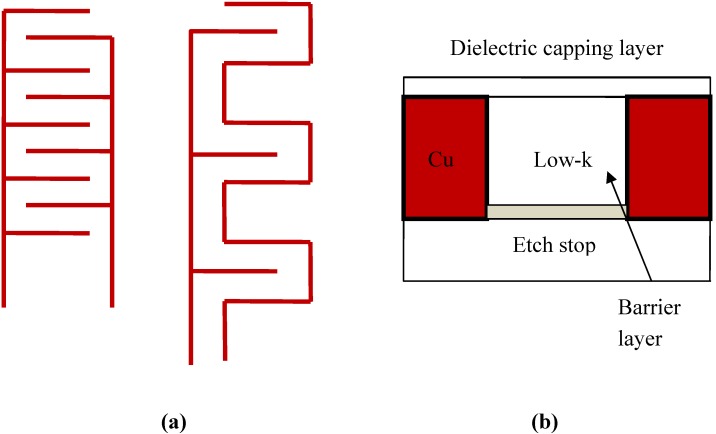
**(a)** Comb-comb and comb-serpentine test structures for TDDB testing; **(b)** Cross sectional schematic of single damascene copper low-k interconnect.

Experimental studies of leakage current and TDDB in Cu low-k interconnects are typically carried out using a single damascene serpentine-comb or comb-comb test structure as shown in Figure 1a. These two test structure geometries provide for a large area for leakage current to flow. The cross sectional view of the test structures is shown schematically in Figure 1b. The test structure consists of an etch stop layer deposited onto the Si substrate and the low-k dielectric layer deposited by plasma enhanced chemical vapor deposition (PECVD). After photolithography of the comb or serpentine pattern in photoresist, trench structures are etched using anisotropic plasma etch. Following resist stripping and cleaning, the Cu barrier metal (e.g., Ta, TaN) and a Cu seed layer are deposited in sequence and the trenches are then refilled completely with Cu using electrochemical deposition. After removal of the Cu overburden by chemical mechanical polishing (CMP), a final top dielectric barrier layer (e.g., SiN, SiCN, SiCO) is deposited by PECVD to prevent Cu migration into the next level of inter-level dielectric.

The top dielectric barrier layer plays an important role in Cu interconnect reliability, because in addition to the leakage and TDDB phenomena discussed in this article, it is also involved in the Cu electromigration issue as discussed in other reviews [31,32]. The effect of the top dielectric barrier layer on the leakage current and dielectric breakdown of Cu-organosilicate glass (OSG) comb capacitor test structures were compared in a series of articles [33,34,35]. In [35], silicon carbide (SiC) and silicon oxycarbide (SiOC) single barrier layers were compared by measuring the current field characteristic at different temperatures for the same OSG intra-level dielectric. The SiOC barrier layer resulted in a substantially lower leakage current at the same field. In addition, the conduction mechanism deduced by fitting was found to depend on the material of the top dielectric barrier layer. For SiC, the leakage current followed a Frenkel-Poole mechanism at high field, while at low field, the conduction was ohmic [35]. For SiOC, the leakage current was better described by the Schottky emission mechanism [35]. This shows that the leakage current primarily flows through the top dielectric barrier layer and the interface between the barrier layer and the OSG [35]. The interface is also significant because during the CMP step, defects are inevitably introduced at the surface of the low-k material [36]. Similar conclusions to the above were drawn for bi-layer top barrier layers comprising amorphous SiC/amorphous silicon carbon nitride (SiCN) [33]. As will be seen in Section 7, the *E^1/2^* low-k dielectric reliability models make explicit use of the leakage through the top dielectric barrier layer in its formulation of the TDDB mechanism.

## 3. Intrinsic Low-k TDDB Models

### 3.1. Lloyd Model

**Figure 2:**
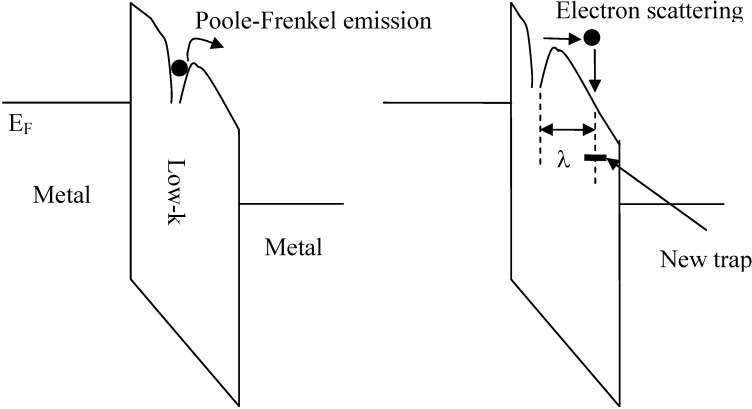
Energy band diagram, illustrating the defect generation process due to inelastic scattering of energetic electrons in Lloyd’s model.

This conceptually straightforward model for low-k TDDB was proposed by Lloyd and co-workers in 2005 and is sometimes referred to as the $\left(1/E+\sqrt{E}\right)$ model [20]. The key idea in the Lloyd model is that damage in the low-k dielectric is caused by energetic electrons. After injection, the electron is accelerated by the electric field in the low-k dielectric and acquires energy. After the electron has traversed a certain distance within the dielectric, it will undergo a scattering event and all the energy that it has acquired from the field up to that point will be dissipated. If the electron has more than a certain threshold energy, a new defect or trap will be generated, (Figure 2). The accumulation of defects in the dielectric eventually leads to TDDB.

Several assumptions are made in the Lloyd model. First, electrons are injected into the low-k dielectric by the Poole-Frenkel mechanism. Second, the electron path length to the scattering site within the dielectric follows an exponential distribution. The third assumption is that there exists a threshold energy for defect generation within the dielectric that is constant with respect to space and time. Finally, it is assumed that the time to breakdown is dependent on the rate at which defects are generated in the low-k dielectric. The Lloyd model does not make any assumptions about the following: (i) the low-k dielectric material being considered; (ii) the nature of the defects caused by the energetic electron; and (iii) the way in which generated defects lead to eventual breakdown.

The rate of defect generation is proportional to the product of the injected electron current and the probability that when the inelastic scattering event occurs, the electron has more energy than the threshold energy for defect generation. The latter can be written using the second assumption as [20]:

(3) $$P\left(\lambda >\lambda_{t}\right)=\text{exp}-\left(\frac{\lambda_{t}}{\mu }\right)$$

In Equation (3), λ is the electron path length; λ*_t_* is the path length needed to acquire the threshold energy *E_t_* and μ is the mean free path of the electron in the low-k dielectric. By using the relationship γ*_t_ = E_t_/qE*:

(4) $$P\left(\lambda >\lambda_{t}\right)=\text{exp}-\left(\frac{E_{t}}{\mu qE}\right)$$

It is assumed that accumulation of defects up to some threshold number *N_f_* will result in dielectric failure at time TTF. Thus, the product of the Poole-Frenkel current Equation (2) and Equation (4) can be integrated from *t* = 0 to *t* = TTF. The time to dielectric failure is predicted to be given by the following equation:

(5) $$TTF=\frac{\left(N_{f}-N_{0}\right)}{AE}\text{exp}\left(-\gamma \sqrt{E}+\frac{E_{t}}{\mu qE}\right)$$

In Equation (5), *N_0_* is the number of pre-existing defects, *A* and γ are the parameters in the Poole-Frenkel equation. Their definition can be found in [20]. The *E^1/2^* dependence in Equation (5) is due to the Poole-Frenkel injection mechanism, while the *1/E* dependence within the exponential function arises from the exponential probability distribution function in Equation (4). At low fields, the *1/E* dependence will predominate.

Experimental verification of the Lloyd model was demonstrated using a total of 20 inter-digitated comb capacitor test structures with an inter-level dielectric having k = 2.3 [37]. Multiple samples of each test structure were stressed at different electric fields and the resulting TTF were plotted according to a single lognormal distribution. The median time to failure spans some seven orders of magnitude for the range of electric fields applied. By using Equation (5) and treating *B*, γ and α as fitting variables, a good fit to the model was obtained [37].

### 3.2. 1/E Model

**Figure 3:**
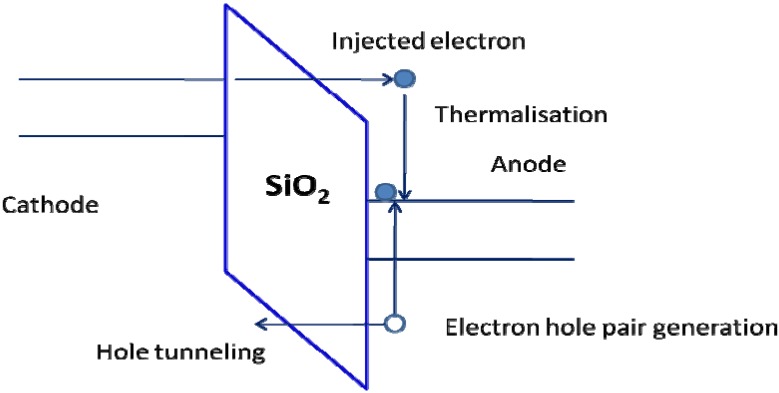
Energy band diagram illustrating the anode hole injection mechanism in *1/E* model.

The *1/E* model was first proposed by Chen *et al.* [21] to explain TDDB in thin gate oxides. A modified form of this model appeared in 1994 [38]. This dielectric failure mechanism is illustrated by the energy band diagram in Figure 3. In this model, one electrode is a metal while the other electrode is a semiconductor. During voltage stressing, a high field develops across the oxide and electrons tunnel into the oxide from the cathode by Fowler-Nordheim tunneling. When these energetic electrons arrive at the anode, they will thermalize and their energy is used to generate holes in the anode. Those holes which are able to surmount the energy barrier at the valence band are then injected back into the dielectric because of the direction of the applied field. This process is called anode hole injection or sometimes as substrate hole injection [17,38]. Since holes have a greater effective mass than electrons, they are more easily trapped within the oxide layer. As a result, positive oxide trapped charge will build up over time near the cathode. This will further increase the electric field near the cathode and lead to more Fowler-Nordheim injection of electrons into the oxide. Eventually, a positive feedback loop will develop and dielectric breakdown will ensue.

The reason for invoking the anode hole injection mechanism is the lack of impact ionization in the oxide. As a result, the type of positive feedback in the oxide field runaway model simply cannot take place and an alternative mechanism is necessary. According to the *1/E* model, the time to breakdown *TTF* is given by the following equation [17,38]:

(6) $$TTF\propto \text{exp}\left(\frac{\beta }{E}\right)$$

where β is the field acceleration parameter for this model. The *1/E* dependence in Equation (6) is due to the Fowler-Nordheim tunneling equation and a semi-logarithmic plot of TTF *versus* the oxide electric field should yield a hyperbola. At low fields, the prediction of the TTF by the *1/E* model tends to converge with that of the Lloyd model.

Since one of the electrodes in the *1/E* model must be a semiconductor, it is questionable whether the *1/E* model can be applied to a damascene Cu low-k interconnect structure. However, as will be elaborated in Section 5, a recent interconnect TDDB study involving Cu/SiO_2_/Si capacitors has yielded results that are consistent with the *1/E* model. This result is somewhat unexpected, because for gate oxides, it has been shown that the *1/E* model is unable to predict correctly the *TTF* at very low fields.

### 3.3. Thermochemical E Model

The themochemical model was originally developed by McPherson and co-workers to predict the time to breakdown for thin gate oxides [18,22]. However, in recent years, it had also been applied to TDDB data from low-k dielectrics. The key concept in the thermochemical model is that TDDB occurs as a result of electric field induced breakage of weak chemical bonds in the dielectric network so that new defects called traps are generated. When a sufficient density of traps is generated in the dielectric, a conductive percolation path linking the two electrodes will be formed and a large increase in current occurs [39]. The silicon oxide layer is irreversibly damaged by thermal effects from this current surge.

**Figure 4:**
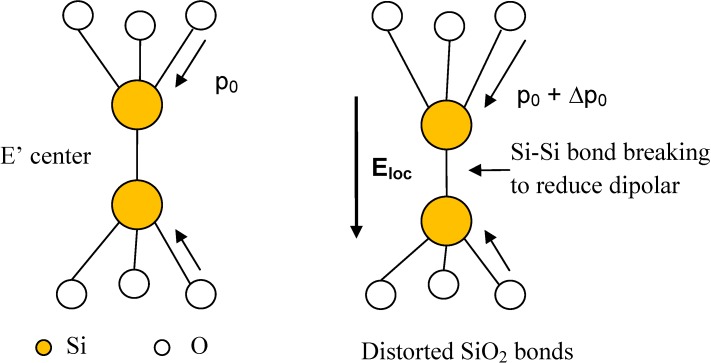
Schematic diagram of the Si-Si bond breaking process of the E’ center in SiO_2_ under voltage stress. Each Si-O bond has a permanent dipole moment *p_0_*. An applied field distorts the Si-O bonds in the O_3_Si-SiO_3_ unit and makes it energetically favorable to break the Si-Si bond.

Since silicon oxide is an amorphous insulator, there is no long-range order in the SiO_2_ network [22]. However, a basic structural unit, namely the tetrahedral SiO_4_ molecular unit, can be identified. In SiO_4_, the four oxygen atoms are located at the vertices of a regular tetrahedron and the Si atom is situated at the centre of the tetrahedron. The bond angle between the two covalent bonds in O-Si-O is always 109.5°. On the other hand, adjacent SiO_4_ molecular units are linked by Si-O-Si bonds via the oxygen atoms at the vertices. As a result of the lack of long-range order, the bond angle can range from 120°–180° [22]. If the bond angle is near the extremes of this range, the bonds will be strained and a defect called the E’ centre is formed instead. The E’ centre (Figure 4) is basically an oxygen vacancy defect and consists of two Si atoms (each bonded to three oxygen atoms) linked by a bridging covalent bond [22]. The main hypothesis in the thermochemical model is that under electric field stress, the Si-Si bonds in the E’ centre of the SiO_2_ will be broken and when a sufficient number of such bonds have been broken, oxide TDDB will occur [22]. The reason why bond breakage should occur at the E’ centre can be seen by comparing bond energies. In [22], the single bond energies of Si-Si and Si-O were calculated using Pauling’s electronegativities of atoms. For Si-Si, only the covalent bond energy contribution is present because of identical atoms and a value of 1.8 eV was found [22]. For Si-O, the significantly different electronegativities of Si and O result in an ionic as well as a covalent component of the bond energy and the total bond energy for Si-O is 5.4 eV [22]. Thus, it is much harder to break the Si-O bond than the Si-Si bond.

The kinetics of the Si-Si bond breakage is assumed to follow a first order reaction in the thermochemical model. This means the rate of Si-Si bond breakage is proportional to the number of such weak bonds *N(t)* available for breakage. The rate equation is written as [22]:

(7) $$\frac{dN}{dt}=-KN(t)$$

here, *K* is the rate constant and is assumed to be given by [22]:

(8) $$K=\upsilon_{0}\text{exp}\left(-\frac{\Delta H_{0}-pE_{loc}}{k_{B}T}\right)$$

in this equation, υ*_0_* is the frequency at which the E’ centre interacts with the oxide network; Δ*H_0_* is the activation energy (or enthalpy) for the breaking of the Si-Si bond. *E_loc_* is the local electric field and *p* is the permanent dipole moment of the SiO_3_ molecular unit. It should be noted that due to the significant difference in electronegativity between Si and O, the Si-O bond is in fact highly polar with 51% ionic character and thus possesses a permanent dipole moment even in the absence of any applied field [40]. The term *pE_loc_* inside the exponential function represents the amount of lowering in the activation energy for bond breakage when an external field is applied. This is because the local electric field is related to the applied field by *E_loc_ = (1+Lχ)E* where *L* is the Lorentz factor (1/3 for SiO_2_) and *χ* is the electric susceptibility (2.9 for SiO_2_) [22].

The reason why Δ*H_0_* is reduced by *pE_loc_* can be seen by considering Figure 4. Here, the electric field is applied along the axial direction of the E’ centre. For this field direction, the dipole moments of the upper SiO_3_ molecular unit will be distorted within the constraints of the network towards being parallel to the applied. The energy of the upper unit *U_u_* is given by [22]:

(9) $$U_{u}=u_{0}-pE_{loc}-\frac{1}{2}\alpha {E_{loc}}^{2}$$

For the lower unit, the corresponding expression for the energy *U_l_* is [22]:

(10) $$U_{l}=u_{0}+pE_{loc}-\frac{1}{2}\alpha {E_{loc}}^{2}$$

*u_0_* is the bond energy of the SiO_3_ molecular unit and α is the polarizability of the unit; the term pE_loc_ is the energy of the permanent dipole in SiO_3_. By comparing Equations (9) and (10), it can be seen that the anti-parallel lower unit has more energy than the upper unit, and this can be reduced by breaking the Si-Si bond. It is suggested that this energy is used to reduce the activation energy for the breaking of the Si-Si bond as indicated in Equation (8). The third terms in Equations (9) and (10) are the energy of the induced dipole moments in the SiO_3_ molecular units because of the applied field. The induced dipole moment can involve displacement of the Si and O atoms as well as the displacement of the electron cloud. They have the same sign in Equations (9) and (10) because the induced dipole moments are independent of the orientation of the SiO_3_ units. In [22], it is shown that the energy of this term is much smaller than the energy of the permanent dipole moment, and so in Equation (8), only the *pE_loc_* term is included in the exponential. By introducing *a = (1 + Lχ)p* in Equation (8) and integrating the rate Equation (7) from *t* = 0 to *t* = TTF, one obtains [22]:

(11) $$TTF=A\text{exp}\left(\frac{\Delta H_{0}-aE}{k_{B}T}\right)$$

the term *a/k_B_T* is also generally known as the acceleration parameter during bias temperature stress experiments. The pre-exponential factor *A* is given by:

(12) $$A=\frac{1}{\upsilon_{0}}\text{ln}\left[\frac{N(0)}{N(t=TTF)}\right]$$

Equation (11) predicts that *ln(TTF)* should vary linearly with the applied electric field *E* with a negative gradient given by the acceleration parameter. By extracting the acceleration parameter from measured TDDB data, the effective dipole moment *a* can be found [41]. When the thermochemical model was applied to gate oxide data [42], an effective dipole moment of ~0.7 qnm was obtained by this method [22]. For interconnect TDDB, a higher effective dipole moment of ~1.3 qnm was found [43]. By using the Mie-Gruniesen potential, McPherson showed that the effective dipole moment of 1.3 qnm was associated with a strained Si-O bond [41].

The thermochemical model is capable of fitting low-k TDDB data at high fields. However, in general, extrapolation to lower fields using this model will tend to give the most conservative predictions. This will be discussed in Section 5.

### 3.4. Haase Model

In the models discussed thus far, the main goal is to develop a predictive model that relates the TTF to the electric field and temperature. When applied, the resulting equations enable the TDDB lifetime at low field conditions to be extrapolated from data obtained at higher field and higher temperature conditions. The Haase model is unique in that it does not aim to develop a TTF relationship on the grounds that some of the microscopic mechanisms used in the previous models lack empirical justification. Instead, it attempts to numerically simulate the low-k leakage current as a function of time and use the time to minimum current (TTMC) as a criterion for dielectric failure [23].

The assumptions made in the Haase model are similar to those found in the Lloyd model. First, TDDB is due to electron current inside the low-k dielectric. Second, only one type of electron trap in the dielectric is considered. When an electron undergoes scattering, all the energy of that electron is dissipated and is used to generate a new trap. As a result, the density of traps within the dielectric increases over time. In addition, the electrons can interact with the optical phonons in the atomic network of the low-k dielectric [23].

The leakage current in the low-k dielectric is found by numerically solving the following three one-dimensional coupled partial differential equations [23]:

(13) $$\nabla \cdot \left[k\varepsilon_{0}\cdot \nabla \cdot V\left(x,t\right)\right]=-q\rho_{total}\left(x,t\right)$$

(14) $$\frac{\partial \rho_{total}\left(x,t\right)}{\partial t}=-\nabla \cdot J_{e}\left(x,t\right)$$

(15) $$\frac{\partial \rho_{trap}(x,t)}{\partial t}=\frac{\left|J_{e}\left(x,t\right)\right|}{l_{scat}}\text{exp}\left[-\frac{E_{a,eff}}{k_{B}T_{e}^{\prime }}\right]$$

Equation (13) is Poisson’s equation and *V(x,t)* is the potential within the low-k dielectric as a function of position *x* and time *t*; ρ*_total_* is the number density of mobile and trapped charges within the dielectric. Equation (14) is the current continuity equation for electrons; *J_e_(x,t)* is the flux of electrons as a function of position and time, and is related to the rate of increase of ρ*_total_*. Equation (15) is the rate equation for the generation of traps within the low-k network; ρ*_trap_* is the number density of traps; *l_scat_* is a characteristic length between scattering events and *E_a,eff_* is the difference between the actual activation energy of the trap and the energy gained by the electron from the field over the distance *l_scat_*. *T_e_’* is likewise an effective temperature and is the weight combination of the wafer temperature *T* and the electron temperature *T_e_*. This linear combination was introduced to take into account the fact that the actual electron energy distribution is neither at thermal equilibrium with the substrate, nor is it characterized by an electron temperature.

In order to solve Equations (13)–(15) for *V*, ρ*_total_* and ρ*_trap_*, the drift-diffusion equation for *J_e_* is needed [23]:

(16) $$J_{e}(x,t)=v_{e}\cdot \left[\rho_{mobile}(x,t)+\rho_{mob\_tunnel}(x,t)\right]$$

Here, υ_e_ is the electron velocity; ρ*_mobile_* is the number density of mobile electrons that are excited thermally from the trap into the conduction band of the dielectric and ρ*_mob_tunnel_* is the number density of mobile electrons that tunnel directly from trap to trap. The bracketed term on the right side of Equation (16) is equal to the difference between ρ*_total_* and the number density of trapped electrons. Hence both ρ*_mobile_* and ρ*_mob_tunnel_* depend on ρ*_total_* and the energy of the trap, *E_trap_*.

If ρ*_total_* and *V(x,t)* are found and *E_trap_* is known, ρ*_mobile_* and ρ*_mob_tunnel_* can be calculated [23]. Substitution into the drift-diffusion Equation (16) would then enable the electron flux and the electron current density at any position within the low-k dielectric to be found as a function of time. In [23], the Haase model was solved for a low-k dielectric layer with a thickness of 120 nm. The parameter values of the model were chosen so that the current density *versus* time curves reproduced the experimental data. The simulated curves decreased slowly with time towards a minimum and then increased afterwards. The TTF for TDDB was defined as 100TTMC. When this estimate of the TTF was plotted as a function of electric field, the TTF is longer than what was predicted by both the *E^1/2^* and *E* models.

## 4. Extrinsic Low-k TDDB Models: Effect of Cu Migration

### 4.1. Cu Drift E Model

It is interesting to note that a similar TTF dependence on electric field to the thermochemical model can be obtained in the limit of large electric fields by considering the drift of Cu^+^ ions inside the dielectric. Cu^+^ ions can diffuse readily in SiO_2_ with a high diffusivity that had been measured at different temperature regimes by various groups [44]. In Si, Cu forms a deep level defect in the band gap of Si that acts as a generation recombination center, and is therefore detrimental to MOS device operation. Cu barriers are therefore deposited prior to Cu deposition to minimize Cu diffusion.

In one of the earliest attempts to develop a quantitative interconnect TDDB reliability model, Wu *et al*. [24] considered the diffusion and drift of Cu^+^ ions in a periodic potential with an external bias. The Cu^+^ ions are thought to be generated from the metal by traps [45]. The basic mechanism of failure is similar to that by Suzumura *et al.*, which is to be discussed next. By considering the continuity equation for Cu^+^ ions and assuming the diffusion component is negligible under TDDB conditions, the following function is obtained [24]:

(17) $$M\left(E\right)=\lambda \left[\text{exp}\left(-\frac{E_{a}-q\lambda E}{kT}\right)-\text{exp}\left(-\frac{E_{a}+q\lambda E}{kT}\right)\right]$$

in the above equation, λ is the periodicity of the potential and *E_a_* is the activation energy that the Cu^+^ ions have to overcome in order to jump to the adjacent potential well. It is also assumed that each Cu^+^ ion can either jump by the distance λ to the left or to the right in a one-dimensional model. The physical interpretation of the function *M(E)* is that it is the net driving force due to drift and is independent of position and time. Thus, the TTF should be inversely proportional to this function. In other words, the predicted TTF according to this model is [24]:

(18) $$TTF=\frac{B\text{ exp}\left(\frac{E_{a}}{k_{B}T}\right)}{\text{exp}\left(\frac{q\lambda E}{k_{B}T}\right)-\text{exp}\left(-\frac{q\lambda E}{k_{B}T}\right)}$$

In the above equation, *B* is a proportionality constant that depends on λ. In the limit of large fields, this TTF relationship reduces to the same form as the thermochemical model with an acceleration parameter given by qλ/κ_B_T:

(19) $$TTF=B\text{ exp}\left(\frac{E_{a}-q\lambda E}{k_{B}T}\right)$$

For experimental verification of their model, Wu *et al*. used the published data of Vogt *et al.* [45] and fitted the data for SiO_x_ and SiN_x_ with Equation (18). The dielectric films were deposited by PECVD using an electron cyclotron plasma source and Cu was deposited by lift-off [45]. For each dielectric, a good fit over a range of electric field and temperature could be obtained with one set of values for *E_a_* and λ.

### 4.2. E^1/2^ Models

In the literature, the *E^1/2^* dielectric reliability model was first proposed for metal-SiN-metal capacitors by Allers [46]. Subsequently, two models that predicted a TTF dependence on the square root of the electric field in low-k dielectrics were reported [25,26]. Unlike the Lloyd model and the thermochemical model which involve intrinsic failure mechanisms, the *E^1/2^* models for low-k dielectrics involve migration of Cu into the low-k dielectric prior to breakdown.

**Figure 5:**
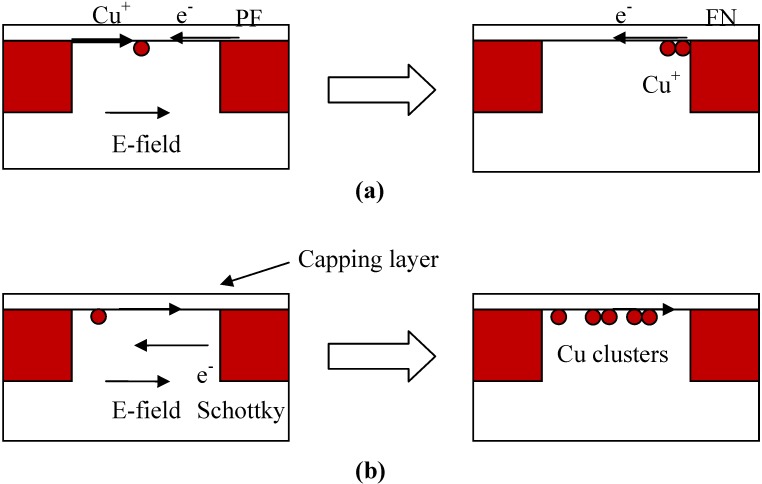
**(a)** Schematic diagram of the process leading to dielectric breakdown in the E^1/2^ model of Suzumura *et al.* [25]; **(b)** Schematic diagram of the process leading to dielectric breakdown in the E^1/2^ model of Chen *et al.* [26].

In the study by Suzumura *et al.* [25], a dielectric capping layer, such as silicon nitride (SiN) and SiC(N,O), was deposited over the Cu interconnects and the intra-level low-k dielectric as shown in Figure 5a. The capping layer provides a leakage path from one Cu conductor to an adjacent conductor and the current conduction mechanism is assumed to be Poole-Frenkel (PF). Accordingly, electrons from the (negative) cathode undergo thermally assisted tunneling from trap to trap in the capping dielectric until the (positive) anode is reached. Under voltage bias stress conditions, Cu^+^ ions from the top of the anode are injected into the interface between the capping layer and the low-k dielectric as shown in Figure 5a and they move towards the cathode. The capping layer low-k interface is preferred, because for Cu, interface diffusion is a faster diffusion pathway [31]. Since the Cu^+^ ions form deep traps in the capping layer dielectric, the electrons moving in the opposite direction will have greater difficulty to de-trap and initially the leakage current decreases. After sufficient time, the Cu^+^ ions arriving at the cathode side will accumulate as a sheet of positive charge and increased band bending occurs in the capping layer dielectric as a result. The leakage mechanism then changes from the Poole-Frenkel to the Fowler-Nordheim mechanism and the leakage current will increase further with time. When the concentration of Cu ions near the cathode exceeds a critical concentration *Q_c_*, TDDB is assumed to occur. Note that this type of mechanism has also been used to explain extrinsic breakdown in gate oxides [47].

The critical concentration *Q_c_* of Cu^+^ ions is proportional to the product of the Cu^+^ ion flux, *J* and the TTF. In [25], the Cu^+^ ion flux was found using the Cu ion concentration *n_i_*, Cu ion mobility and the electric field in the capping layer. For the Cu ion concentration, a Poole-Frenkel type expression was used for the Cu ion transport [25]:

(20) $$n_{i}\propto n_{0}\text{ exp}\left[\frac{q}{k_{B}T}\sqrt{\frac{qE}{\pi \varepsilon_{0}k}}\right]$$

The time to failure is therefore given by [25]:

(21) $$TTF\propto \frac{1}{E}\text{exp}\left[-\frac{q}{k_{B}T}{\left(\frac{q}{\pi \varepsilon_{0}k}\right)}^{1/2}\sqrt{E}\right]$$

In the *E^1/2^* model by Chen *et al.* [26], electrons are considered to be injected from the cathode into the low-k dielectric by Schottky emission (Figure 5b). Those electrons which do not undergo scattering within the low-k dielectric become energetic and can use their energy upon arrival at the anode to generate Cu^+^ ions. These Cu^+^ ions are then injected into the low-k dielectric at the low-k capping layer interface as discussed above and move towards the cathode. Chen *et al.* proposed two mechanisms by which the final breakdown may occur. In the first scenario, the Cu^+^ ions combine with electrons and become neutral Cu atoms. These atoms agglomerate into Cu clusters that over time can coalesce into a metallic bridging short. This hypothesis however, was inconsistent with the results of Lloyd *et al.* [48]. In the second scenario, the Cu atoms by virtue of their size can increase the local strain in the low-k dielectric and facilitate bond breakage. The condition for TDDB can be expressed by a critical concentration of Cu at the capping layer low-k interface. In [26], the concentration of Cu^+^ ions at the interface is determined from the diffusion equation. Since the Cu interconnects are effectively an infinite source of Cu ions, the one-dimensional solution of Fick’s first law subject to this boundary condition is applicable and the solution of the diffusion is described by the complementary error function [26]. By integrating this concentration function with respect to distance, the number of Cu atoms per unit area at the interface at breakdown can be written as [26]:

(22) $$C_{crit}=1.12C_{Cu}\sqrt{TTF.D_{0}\text{ exp}\left[\frac{-E_{D}}{k_{B}T}\right]}$$

Here, *E_D_* is the activation energy for Cu diffusion and *D_0_* is the pre-exponential coefficient of the Cu^+^ ion diffusivity. Equation (22) shows that the critical concentration of Cu at breakdown, *C_crit_* is given by the concentration of Cu^+^ ions generated at the anode *C_Cu_* and the diffusion length of the Cu^+^ ions. *C_Cu_* is assumed to be proportional to the electron current, because these ions are generated by the energy of the energetic electrons. Chen *et al.* assumed a Schottky emission model for the electron current, and the time to failure by TDDB is then given by [26]:

(23) $$TTF\propto \frac{C_{crit}^{2}l_{0}^{2}}{D_{0}A^{*2}T^{4}}\text{exp}\left[\frac{1}{k_{B}T}\left(E_{D}+2\phi_{s}-2\beta_{s}\sqrt{E}\right)\right]$$

where *l_0_* is the total wire length; *φ_s_* is the barrier height and *β_s_* = (q^3^/4πκε_0_)^1/2^.

Experimental verification of the two *E^1/2^* models was carried out at wafer and module level using comb-serpentine and comb-comb capacitor structures with line-to-line spacing of 100–200 nm [25,26]. The low k dielectrics studied included dense SiOF (k = 3.7) [25], SiOC (k = 3.0) [25] and SiOCH (k = 3.0) [26]. The dielectric capping layers were SiN (k = 7.0) and SiC(N,O) (k = 4.4–4.8) [25]. The test structures were stressed at different electric fields and temperature until dielectric breakdown. From the cumulative failure distribution for each test condition, the characteristic Weibull 63.2% time to breakdown *t_63.2_* was extracted. By plotting the *t_63.2_* *versus* electric field, the *E* model was found to be a poor fit at low fields. However, the *E^1/2^* model was able to predict the observed *t_63.2_* at the lowest electric fields. Additional evidence supporting the *E^1/2^* model was obtained by measuring the temperature dependence of the leakage current at different stress times and field dependence of the activation energy [25]. In a separate study, Yiang *et al.* also presented experimental data supporting the *E^1/2^* model for low-k TDDB involving SiOCH [49].

### 4.3. E^2^ Model

The *E^2^* model was proposed by Achanta and co-workers in 2007 [27]. Like the *E^1/2^* models, it assumes that Cu^+^ ion diffusion and drift play a major role in the TDDB of low-k dielectrics. During electrical stressing, Cu^+^ ions migrate into the low-k dielectric from the anode. After sufficient time has elapsed, a sheet of Cu^+^ ions will accumulate at the cathode that will result in an increase in the electric field near the cathode. For the diffusion/drift of Cu ions, a mass transport model was developed, in which the coupled nonlinear Poisson equation and continuity equations were solved to yield the Cu^+^ ion concentration and the potential within the dielectric [27]. This is the difference from the modeling approach of Chen *et al.* [26]. In addition, the boundary condition chosen is that the Cu^+^ ion current density is zero at the cathode. This condition results in the accumulation of Cu^+^ ions over time. From the simulation results of this mass transport model, a function called *f(C_e_, T, E_app_)* can be computed. The function *f(C_e_, T, E_app_)* gives the time taken for the electric field at the cathode to increase to the breakdown field *E_bd_* and is a function of the Cu ion solubility in the low-k dielectric, *C_e_*, the absolute temperature, *T* and the applied field, *E_app_*.

Instead of increased Fowler Nordheim tunneling [25], it is assumed that the enhanced field at the cathode will eventually lead to bond breakage at defects in the dielectric as in the thermochemical model. However, it was found that if the exponential function in the thermochemical model were used without modification, then the measured TTF cannot be fitted by one set of fitting parameters only for experimental data collected from test structures at different temperatures. The proposed TTF function is written instead as [27]:

(24) $$TTF=A\text{exp}\left(\frac{E_{a}-\gamma E_{app}^{2}}{k_{B}T}\right)f\left(C_{e},T,E_{app}\right)$$

where the activation energy *E_a_* is reduced by the energy of the induced dipole moment of the Si-O bonds [see quadratic term in Equation (9)]. When this equation is applied to experimental data collected at different temperatures, only one set of fitting parameters is needed to fit all data satisfactorily. It was argued that when there is Cu present in the low-k dielectric, the induced dipole moment energy could be more significant than the permanent dipole moment energy.

For experimental verification, Achanta *et al.* made use of the published experimental data of Hwang *et al.* on Cu/SiO_2_/Si capacitors [50]. The function *f(C_e_, T, E_app_)* was calculated for SiO_2_ and Equation (24) was used to fit the experimental data of Hwang *et al.* from 150 °C to 250 °C. A good fit could be obtained for all data points with one set of parameter values for *A*, λ and *E_a_* [50].

### 4.4. Effect of Cu Migration on Porous Low-k Dielectric Reliability

The migration of Cu is especially relevant to porous low-k dielectrics which are now used for Cu interconnects. In an early study involving porous SiOC and porous MSQ [43], Ogawa and co-workers proposed a percolation model for the TDDB in these materials. The dielectric is considered to be composed of cells, some of which are defective. The pores that are present in the as-deposited material are represented as defective cells. During electrical stressing, additional defective cells will be generated. When the average fraction of defective cells λ, reaches a critical value λ*_bd_*, for a dielectric with porosity *P*, a percolation path is formed between two Cu electrodes. The resulting wavefunction overlap causes breakdown to occur [39]. It is assumed that the breakdown field *E_bd_* occurs when λ is equal to λ*_bd_*, so that [43]:

(25) $$\frac{{\left(E_{bd}\right)}_{with\_porosity}}{{\left(E_{bd}\right)}_{without\_porosity}}=\frac{{\left(\lambda_{bd}\right)}_{with\_porosity}}{{\left(\lambda_{bd}\right)}_{without\_porosity}}=1-\frac{P}{{\left(\lambda_{bd}\right)}_{without\_porosity}}$$

Equation (25) predicts that the breakdown field should decrease with porosity. The fraction of capacitors that undergoes TDDB, *F_bd_* is given by the Weibull relation [43]:

(26) $$\text{ln}\left\{-\text{ln}\left[1-F_{bd}(\lambda )\right]\right\}=n_{bd}\text{ln}(\lambda )+\text{ln}(N_{col})$$

In Equation (26), *n_bd_* is the number of cells in the percolation path and *N_col_* is the number of columns in the capacitor. In a related study [51], Hwang *et al.* used capacitance voltage and positronium annihilation lifetime spectroscopy (PALS) to study the TDDB of a series of porous MSQ dielectrics with porosity from 0% to 40%. When the porosity reached 20%, there was a significant drop in the TTF characterized by the *t_63.2_* parameter of the Weibull distribution. At the same time, there was a rise in the Weibull slope β. The flatband voltage, *V_FB_*, of the capacitance voltage curves also showed an increased shift. These observations were interpreted as a change from a closed pore to an interconnected pore structure. The migration of Cu changed from bulk diffusion to surface diffusion, which is faster, and made the dielectric more vulnerable to breakdown.

The assumption of Cu ion drift in the *E^1/2^* and *E^2^* models was tested experimentally by He *et al.* recently [52]. By using secondary ion mass spectrometry (SIMS) and capacitance voltage characterization on Cu/p-SiOCH/Si structures, it was found that Cu atom diffusion already occurred after deposition. Although Cu penetration increased with annealing, bias temperature stress does not affect the SIMS profile and there was no *V_FB_* shift. This result calls into question the key assumption in the *E^1/2^* and *E^2^* models. Finally, in a very recent study [53], Lin *et al.* studied the effect of Cu surface roughness after chemical mechanical polishing (CMP) on the TDDB of ultra low-k dielectrics. The surface roughness correlated strongly with TDDB. In samples where Cu surface roughness was high, cracking of the metal capping layer occurred and Cu penetrated into the interface between the ultra low-k and capping layer. More ultra low-k polishing and increased deionized water dilution of the post CMP cleaning could reduce Cu surface roughness [53].

## 5. Validation of Low-k Dielectric Reliability Models

Since various models currently exist in the literature regarding the reliability of low-k dielectrics, it is necessary to carry out systematic experiments that compare the various predictive models by the same set of experimental data over a wide range of electric fields. This can help to establish which model is valid for predicting low-k TDDB at operational conditions. Experiments of this kind had previously been conducted at low fields and by long-term stressing for thin gate oxides to validate the *E* and *1/E* models [17]. Initial work of this kind had been carried out by Croes and Tokei using serpentine-comb and comb-comb test structures fabricated from SiOCH with k = 2.5 and 25% porosity [54]. The actual line-to-line spacing was measured by transmission electron microscopy to be 105 nm and 45 nm. During TDDB testing, electric fields in the range of 2.2 MV/cm to 5.2 MV/cm were applied to the test structures and the cumulative failure distributions were collected for each applied field. By using the median time to failure derived from the cumulative distributions of the three highest fields for statistical fitting, the median time to failure and the 95% prediction interval at the lowest field were extrapolated using the *E*, *E^1/2^*, *E^n^* (power law) and the *1/E* models. When the various predictions were compared with experimental data collected at the lowest field, it was found that both the *E* and *E^1/2^* models were overly conservative and underestimated the TDDB lifetime of these samples. These investigators, however, were unable to identify a dielectric reliability model that is capable of predicting the TTF for their samples.

More recently, Zhao and co-workers adopted an earlier approach for validating the interconnect reliability models [55]. For this study, metal-insulator-semiconductor (MIS) capacitors were used instead of the inter-digitated comb or serpentine test structures. Cu metal was deposited directly onto SiO_2_ grown on a n-type Si substrate. The rationale for using MIS structures is that in order to validate any of the available models, it is essential to have measured (not extrapolated) TDDB experimental data at low fields [55]. However, in conventional comb or serpentine test structures, the use of Cu barrier metals can greatly reduce the Cu diffusion rate into the dielectric materials and renders it impractical to measure the TTF at low fields within a reasonable time span [55]. In addition, electric field enhancement at the corner regions of damascene test structures may cause TDDB. This issue can be avoided by using MIS structures. A further benefit of the MIS structure is that process-related defects which can broaden the TTF statistical distribution can be avoided [55].

**Figure 6:**
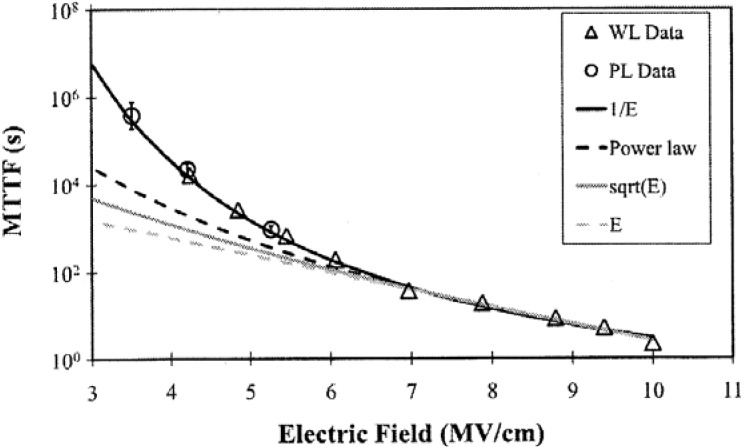
Experimental validation of four proposed dielectric reliability models using Cu/SiO_2_/Si capacitors. Reprinted with permission from reference [55]. Copyright (2011) by the American Institute of Physics.

In the experiment by Zhao *et al.*, Cu/SiO_2_/Si test structures were tested at electric fields ranging from 3.5MV/cm to 10MV/cm. By using only the TTF data at high fields, extrapolation to low fields was performed using the *E, 1/E, E^1/2^* and power law models. Only the *1/E* model correctly predicts the low field experimental data which have small error bars (Figure 6) [55]. It was concluded that for the Cu/SiO_2_/Si system, the *1/E* model provides the best prediction of the TTF for TDDB. However, further research is needed for evaluating the Lloyd model and the *E^2^* model which were not studied and to apply this methodology to evaluate low-k dielectrics.

## 6. Soft Breakdown in Porous Ultra Low-k Dielectrics

**Figure 7:**
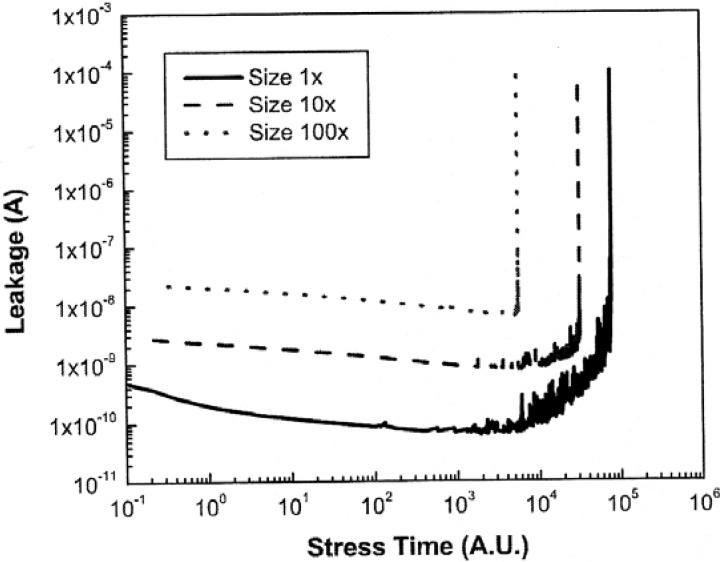
Current voltage characteristics of soft breakdown in Cu ultra low-k test structures with three different test structure sizes (1×, 10×, 100×). Reprinted with permission from Reference [56]. Copyright (2010) by the American Institute of Physics.

As the semiconductor industry introduces porous ultra low-k dielectrics for state–of-the-art ICs, the study of the TDDB phenomenon is further complicated by reports of soft breakdown (SBD) which often precedes the onset of HBD. SBD (also known as quasi-breakdown) was first observed over a decade ago in ultrathin (< 4 nm) gate oxide films [17]. Although SBD is relatively well understood for gate oxides, the study of the SBD phenomenon has only just begun for interconnect dielectrics. Croes and Tokei reported observation of SBD in porous SiOCH at low fields in test structures with a nominal line-to-line spacing of 50 nm [54]. In one of the first detailed studies on this phenomenon [56], Chen and Shinosky studied chemical vapor deposited (CVD) porous SiOCH with k = 2.4. Serpentine-comb and comb-comb Cu damascene structures with an inter-line spacing of 10–50 nm were characterized by constant voltage stress (CVS) and constant current stress (CCS) [56]. These investigators found that during CVS, most samples showed a small increase in leakage current and leakage current noise prior to HBD where the current increased abruptly. An increase in current noise was also observed for SBD in ultrathin gate oxides [17]. More interestingly, SBD was found to occur more readily in smaller test structures and at lower stress voltages (see bottom curve, Figure 7). In addition, by using CCS, it was possible to observe only SBD and completely avoided HBD because of self-limiting power dissipation [56]. These observations have important implications for future technology nodes because with device scaling and reduced power supply voltages, the incidence of SBD should become more common.

By performing cross-sectional transmission electron microscopy, Chen and Shinosky attributed SBD in ultra low-k dielectrics to Cu penetration resulting from poor liner integrity. The etched surface of a porous low-k surface has a greater surface roughness and thus adversely affects the liner uniformity [56]. The migration of Cu into the pores of the dielectric leads to increased current leakage and current fluctuations.

It should be noted, however, that Cu migration may not be the only cause of SBD. In [57], Matz and Reidy reported that after cleaning by pure supercritical carbon dioxide (SC-CO_2_), there was an improvement in the breakdown field strength in SiOCH (k = 2.5). Since the role of the SC-CO_2_ is to remove residual chemicals in the SiOCH, these chemicals can also be responsible for SBD.

## 7. Conclusions

In this article, the research on low-k TDDB between 2003 and 2012 has been reviewed. For this complex phenomenon, there is as yet no consensus on the physical failure mechanism. The main dielectric reliability models have not been thoroughly tested for different low-k materials and at low electric fields. The models of Wu *et al.* [24] and Achanta [27] had only been applied to SiO_2_. For the other models [20,25,26], only SiOCH had been studied experimentally. As a result, there is currently a large number of dielectric models all of which aim to predict the time to breakdown at electric fields and temperatures at use conditions from accelerated test data. In order to narrow down the number of models, it may be useful to carry out a collaborative round-robin type experiment in which Cu low-k test structures fabricated at one laboratory is distributed to different research groups for testing and model verification.

Finally, with the transition to porous low-k materials in future technology nodes, it may become necessary to reconsider the operational definition of breakdown in interconnect dielectric materials. This is due to the effects of Cu migration and the prevalence of SBD. In addition, the notion of a uniform field inside the ultra low-k dielectric may have to be modified to take into account electric field enhancement effects in interconnect dielectric reliability models [16]. Field enhancement could be due to pattern line edge roughness [58] and porosity [51,52,59]. The former is due to limitations of the lithography and pattern transfer process and does not scale with feature size. Since it has the effect of further reducing the TTF, its effect will need to be quantified and built into dielectric reliability models in future. The separation of field enhancement due to test structure structural effects and porosity is likely to be a key focus of future research.
